# Could KIM-1 and NGAL levels predict acute kidney injury after paracentesis? – preliminary study

**DOI:** 10.1080/0886022X.2020.1801468

**Published:** 2020-08-18

**Authors:** Marta Pietrukaniec, Maciej Migacz, Agnieszka Żak-Gołąb, Magdalena Olszanecka-Glinianowicz, Jerzy Chudek, Jan Duława, Michał Holecki

**Affiliations:** a Department of Internal, Autoimmune and Metabolic Diseases, Medical Faculty in Katowice, Medical University of Silesia, Katowice, Poland; b Department of Pathophysiology, Medical Faculty in Katowice, Medical University of Silesia, Katowice, Poland; c Department of Internal Diseases and Oncological Chemotherapy, Medical Faculty in Katowice, Medical University of Silesia, Katowice, Poland; d Department of Internal and Autoimmune Diseases, School of Health Science, Medical University of Silesia, Katowice, Poland

**Keywords:** Acute kidney injury, liver cirrhosis, NGAL, KIM-1, paracentesis

## Abstract

**Background:**

Kidney dysfunction is a common complication in patients with severe liver cirrhosis. There is a need for discovery and validation of novel biomarkers for earlier AKI detection. The aim of this study was to determine if tubular injury markers: NGAL and KIM-1 could be helpful in the early diagnosis of AKI in patients undergoing therapeutic paracentesis.

**Methods:**

This preliminary study included 24 adult patients diagnosed with liver cirrhosis who had been hospitalized due to massive ascites requiring paracentesis. Pre- and post-paracentesis plasma samples were taken from each patient and biomarkers were measured.

**Results:**

Before paracentesis, the levels of serum and urinary NGAL were similar between patients and controls; while urinary KIM-1 was markedly increased in liver cirrhotic patients (0.76 vs. 0.24 ng/ml; respectively). Although urinary NGAL levels in AKI patients were 5-time greater than in non-AKI subgroup, the difference did not reach statistical significance (13.2 vs 1.5 pg/mL, *p* = 0.06). Serum NGAL level, post-procedure, was 3 times greater in AKI subgroup.

**Conclusion:**

Kidney injury markers, especially serum NGAL, may be useful for the early detection of AKI. However, further research is required to determine if biomarkers of kidney injury may help identify patients with cirrhosis who would most likely benefit from early AKI prevention and treatment.

## Introduction

Kidney dysfunction is a common complication in patients with severe liver cirrhosis related to the abnormal hemodynamics of systemic and splanchnic arterial vasodilatation and extrahepatic vasoconstriction [1]. Acute kidney injury (AKI) is mainly pre-renal and largely asymptomatic. The diagnosis is currently based on functional biomarkers such as serum creatinine or decreased urine output, both of which have quite low sensitivity [2]. Creatinine fluctuation early in the course of AKI is frequent and difficult to interpret; it may take several days to resolve into a definite trend demonstrating progression [3]. AKI develops in approximately 20% of hospitalized patients with liver cirrhosis and is associated with increased mortality. The risk of death increases along with the severity and progression of AKI, independent of the model of end-stage liver disease (MELD) score [4–6]. Concurrently, there is a need for discovery and validation of novel biomarkers for earlier AKI detection. Among the most promising tubular biomarkers of AKI, measured in both serum or urine, there are kidney injury molecule-1 (KIM-1) and neutrophil gelatinase-associated lipocalin (NGAL). Some data suggest that they may identify patients with sub-clinical AKI who have an increased risk of adverse outcomes [7].

KIM-1, a marker of proximal tubular injury, is a transmembrane protein upregulated by renal ischemia. It is barely detectable in the urine of healthy subjects but substantially increased in patients with acute tubular necrosis (ATN). Of note, no increase was observed in patients with prerenal azotemia, urinary tract infection and chronic kidney disease [8,9]. In a multivariate model, a one-unit increase in normalized KIM-1 was associated with a greater than the 12-fold risk for the presence of ATN [9]. KIM-1 in urine increases already within the first hours after toxic or ischemic damage of the renal tubules, whereas the increase in serum creatinine is usually observed later on [10].

Neutrophil gelatinase-associated lipocalin (NGAL) is a 25 kDa protein belonging to the lipocalin superfamily [11,12]. While it may be secreted by various types of human tissues (predominantly by epithelial cells and neutrophils), in the kidneys, NGAL is mostly expressed in the thick ascending limb of Henle loop and collecting ducts of the kidney [13,14]. NGAL seems to be a very sensitive biomarker of kidney injury, because its increase, both in plasma and in urine, occurs already 2 h after the injury, and substantially preceding serum creatinine elevation that occurs around 24–48 h later [10].

Therapeutic paracentesis (especially one in which a large volume is withdrawn) is an important risk factor for AKI, due to intravascular volume depletion and pre-renal dysfunction [15]. This *postparacentesis syndrome*, which occurs in up to 80% of cases when a large volume paracentesis is performed without anticipatory therapeutic volume replacement, was firstly described by Gines et al. in 1988 [16]. While therapeutic paracentesis is the treatment of choice in patients unresponsive to conservative treatment, even though it is associated with the risk of AKI. As the development of AKI correlates with poor prognosis including increased mortality, early diagnosis of AKI is important. This study aimed to determine if tubular injury markers: NGAL and KIM-1 could be helpful in the early diagnosis of AKI in patients undergoing therapeutic paracentesis.

## Materials and methods

This preliminary study included 24 adult patients (12 males and 12 females; age range 34–93) diagnosed with alcoholic (even if currently abstinent) or viral (chronic hepatitis B without a history of antiviral therapy) liver cirrhosis who had been hospitalized at the Department of Internal Medicine due to progressive and painful abdominal distension from massive ascites requiring paracentesis. The diagnosis of decompensated liver cirrhosis was based on clinical presentation and radiological evidence of a shrunken liver with a dilated portal vein, presence of ascites and collaterals. We did not collect detailed information why patients with hepatitis B were not receiving antiviral therapy (decompensated liver cirrhosis is considered as an exclusion criterion for antiviral therapy in the drug program covered by the Polish National Health Fund). We did exclude both active or subclinical infections, hepatocellular carcinoma, and active bleeding. There was a history of upper gastrointestinal bleeding in one-third of the study group. Besides, more than half of the patients (*N* = 14) have had a previous paracentesis. It was obligatory for inclusion that patients undergoing paracentesis had at baseline urine output of >500 mL/day and serum creatinine of ≤1.5 mg/dl.

### Therapeutic paracentesis protocol

Patients and their attendants have explained the need for paracentesis and procedure-related complications. After obtaining written informed consent, the procedure was performed under ultrasound guidance following the standard hospital protocol with close monitoring of pulse, blood pressure and respiratory rate. The fluid was removed at variable rates but not exceeding >5 L daily. We did not observe hemodynamic instability during or after the procedure. Eighteen healthy adult subjects (6 males and 12 females, age range 30–70 years) were enrolled as the control group. Patients and control characteristics are presented in Table 1.

**Table 1.** Characteristics of patients with liver cirrhosis and apparently healthy controls.

	Liver cirrhosis group	Controls	
Parameters	*N* = 24	*N* = 18	*p*-value
Age (years)	68 (61–75)	42 (35–49)	<0.001
Sex (Men/Women)	11/13	4/14	0.12
The causes of liver cirrhosis (n)			
Inflammatory	2	–	
Alcoholic	11	–	
Mix	2	–	
Unknown	9	–	
Child-Pugh score (n)			
Class A	1	–	
Class B	12	–	0.12
Class C	11	–	
Hb (g/dL)	11.6 (10.4–12.7)	14.3 (13.9–14.7)	<0.001
PLT (10^3^/µL)*	178 (114–282)	305 (253–370)	<0.01
WBC (10^3^/µL)	10.2 (8.0–12.3)	7.2 (5.9–8.5)	<0.05
ALT (U/L)	24.3 (17.2–31.5)	27.9 (25.2–30.6)	0.39
INR*	1.53 (1.15–1.99)	0.95 (0.80–1.00)	<0.001
Serum protein (g/dL)	58.6 (56.1–61.2)	74.3 (71.7–76.9)	<0.001
Serum albumin (g/dL)	26.6 (24.1–29.2)	42.3 (39.7–44.9)	<0.001
C-reactive protein (mg/L)*	36.9 (20.8–59.0)	<5	<0.001
Serum creatinine (mg/dL)	1.56 (0.92–2.21)	0.86 (0.78–0.94)	<0.01
eGFR (mL/min/1.73m^2^)	65.2 (50.3–80.1)	91.0 (80.3–101.7)	0.06
Serum bilirubin (mg/dL)	3.07 (1.94–4.20)	0.96 (0.89–1.02)	<0.001
Serum NGAL (pg/mL)*	60.8 (40.5–115.6)	75.8 (44.1–115.6)	0.26
Urinary NGAL (pg/mL)*	4.0 (1.2–17.4)	3.9 (1.3–8.2)	0.35
Urinary KIM-1 (ng/mL)*	0.76 (0.21–1.83)	0.24 (0.19–0.41)	<0.01

Data is presented as mean value with 95% confidence interval) or median with quartiles (1–3Q)*. NGAL: neutrophil-gelatinase associated lipocalin. KIM-1: kidney injury molecule 1.

The research protocol was approved by the local Ethics Committee of the Medical University of Silesia in Katowice (KNW/002/KB1/72/I/16/17). All clinical investigation was conducted according to the principles expressed in the Declaration of Helsinki. Signed informed consent was obtained from all subjects who participated in the study.

Patients and controls were subjected to complete history taking along with full clinical examination, and laboratory investigations.Pre- and post-paracentesis (48-72 h) serum creatinine, NGAL and urine KIM-1 were checked. Serum bilirubin, alanine aminotransferase, total protein and C reactive protein were assessed on admission to hospital (as a routine procedure). The serum and urine samples were stored in −70 °C until the time of the assay.

### Laboratory analyses

Serum bilirubin, creatinine, alanine aminotransferase, total protein, albumin and C reactive protein were determined by spectrophotometry (Point Scientific Inc., Michigan, USA) in a single-certified laboratory. Serum and urine total NGAL and urine KIM-1 measurements were performed using commercially available ELISA kit (BioPorto Diagnostics Gentofte, Denmark and BioAssay Works, Ijamsville, DM, USA, respectively) with intraassay coefficients of variation of 7.9%, and the limit of quantification of 0.01 ng/mL.

### Data analysis

Model for End-Stage Liver Disease (MELD) score was calculated according to a standard formula, as follows: MELD = 3.78 × ln [serum bilirubin (mg/dL)] + 11.2 × ln [INR] + 9.57 × ln [serum creatinine (mg/dL)] + 6.43 [17]. Glomerular filtration rate (eGFR) was estimated by the CKD-EPI equation using the baseline creatinine value [18].

AKI was defined as a rise in creatinine of equal or more than 0.3 mg/dL or 50% from baseline as recommended by a working group composed of members of the IAC and ADQI who based this cutoff on Stage 1 of the AKIN criteria [19].

### Statistics

The analysis was performed using MedCalc 18.6 licensed software (MedCalc Software, Ostend, Belgium). Quantitative variables are presented as means with 95% confidence interval or medians with interquartile range (1–3Q), due to non-parametric distribution on numerous parameters. Qualitative variables are presented as absolute values and percentages.

The chi-square test was used for qualitative variables. The inter-subgroup differences were assessed with the Student *t*-test and the Mann–Whitney U-tests, when appropriate. Correlation coefficients were calculated according to Spearman. For all analyses, *p*-value below 0.05 was considered as statistically significant.

## Results

The clinical characteristics of the patients with liver cirrhosis (mostly Child-Pugh class B or C) and apparently healthy controls are summarized in Table 1. Liver cirrhotic patients had lower serum hemoglobin (11.6 vs. 14.3 mg/dL; *p* < 0.001), platelet count (178 vs. 305 × 10^3^/µL; *p* < 0.01); albumin (26.6 vs 42.3 g/dL; *p* < 0.001), and near significantly lower eGFR values (65.2 vs 91.0 mL/min/1.73m^2^; *p* = 0.06, respectively) than the controls. As expected patients with liver cirrhosis had higher serum bilirubin (3.07 vs. 0.96 mg/dL; *p* < 0.001), C-reactive protein (36.9 vs. < 5 mg/L), and INR (1.53 vs. 0.95; *p* < 0.001; respectively) than controls. Before paracentesis, the levels of serum and urinary NGAL were similar between patients and controls; while urinary KIM-1 was markedly increased in liver cirrhotic patients.

There was a strong correlation between serum creatinine and serum (*R* = 0.510; *p* = 0.01) but not urinary (*R* = 0.004; *p* = 0.98) NGAL levels in patient with liver cirrhosis. The association with eGFR was weaker (R = −0.335; *p* = 0.09). There was no association between kidney function measures and urinary KIM-1 levels.

### Post paracentesis changes

All cirrhotic patients had one paracentesis within 3 days period. Median paracentesis volume was 5.1 L. In the postprocedural 48–72 h, one-third of the patients had deterioration of kidney excretory function that met criteria for AKI (Table 2).

**Table 2.** Comparison of cirrhotic patients who developed acute kidney injury (AKI) after paracentesis or who did not.

	AKI	No-AKI	
Parameters	*N* = 8	*N* = 16	*p*-value
Age (years)	70 (62–81)	65 (56–81)	0.83
Sex (men/women)	4/4	7/9	0.78
Hb (g/dL)	11.1 (8.3–13.1)	12.9 (10.6–13.7)	0.24
PLT (10^3^/µL)	183 (130–241)	171 (114–313)	0.83
WBC (10^3^/µL)	8.0 (5.4–11.2)	10.1 (6.1–13.8)	0.53
ALT (U/L)	27.0 (18.5–33.5)	14.5 (9.5–29.5)	0.21
Serum protein (g/dL)	56.5 (52.1–61.0)	58.8 (56.5–64.6)	0.38
Serum albumin (g/dL)	24.5 (20.1–29.0)	26.8 (24.5–32.6)	0.38
INR	1.41 (1.13–1.83)	1.66 (1.17–2.02)	0.49
C-reactive protein (mg/L)	39.4 (23.9–118.8)	36.9 (20.8–55.2)	0.70
Serum creatinine (mg/dL)	1.14 (0.75–1.50)	1.20 (0.55–1.53)	0.79
eGFR (mL/min/1.73m^2^)	54.5 (44.0–91.0)	51.5 (36.6–104.0)	0.88
Serum bilirubin (mg/dL)	2.77 (1.35–3.95)	2.34 (1.04–4.77)	0.93
Serum NGAL (pg/mL)	70.1 (43.1–157.9)	52.4 (34.7–97.1)	0.32
Urinary NGAL (pg/mL)	11.3 (3.3–57.8)	1.7 (1.0–6.2)	0.11
Urinary KIM-1 (ng/mL)	0.97 (0.23–2.91)	0.65 (0.20–1.35)	0.42
Child-Pugh score (*n*)			
Class A	0	1	
Class B	4	8	0.76
Class C	4	7	
MELD score (pts)	21.3 (17.5–30.2)	17.4 (10.8–27.9)	0.32
MELD scor*e* > 20 pts (*n*)	4 (50%)	7 (44%)	0.78
Total paracentesis volume (L)	6.0 (4.8–8.7)	4.8 (2.9–5.9)	0.08
After paracentesis			
Serum creatinine (mg/dL)	1.65 (1.05–3.55)*	(0.60–1.50)	<0.05
Serum NGAL (pg/mL)	149.5 (59.7–216.3)**	45.2 (28.9–77.0)	0.01
Urinary NGAL (pg/mL)	13.2 (1.7–66.2)	1.5 (1.0–7.0)	0.06
Urinary KIM-1 (ng/mL)	0.62 (0.20–0.83)	0.43 (0.26–1.13)	0.93

Data is presented as median with quartiles (1–3Q).

*p < 0.05, **p = 0.01.

Although urinary NGAL levels in AKI patients were 5-time greater than in non-AKI subgroup the difference did not reach statistical significance (*p* = 0.11) (Table 2).

**Figure 1.**
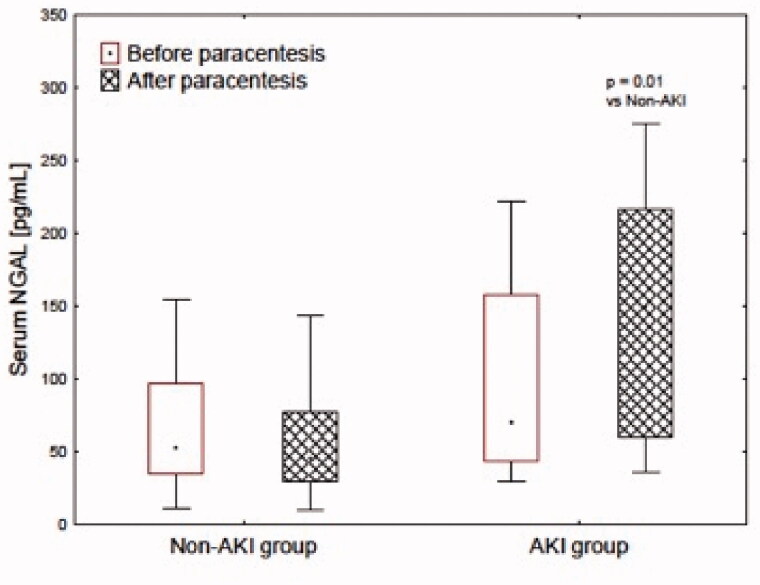
Serum NGAL concentration before and after paracentesis in AKI and non-AKI group.

Serum NGAL level, post-procedure, was 3 times greater in AKI subgroup (Table 2). In the non-AKI group, the values were stable (Figure 1). As a consequence, post-procedural values of serum NGAL were greater in AKI, then the non-AKI subgroup. Urinary NGAL (Figure 1) and KIM-1 values were unaltered in the second examination in both AKI and non-AKI subgroups.

**Figure 2.**
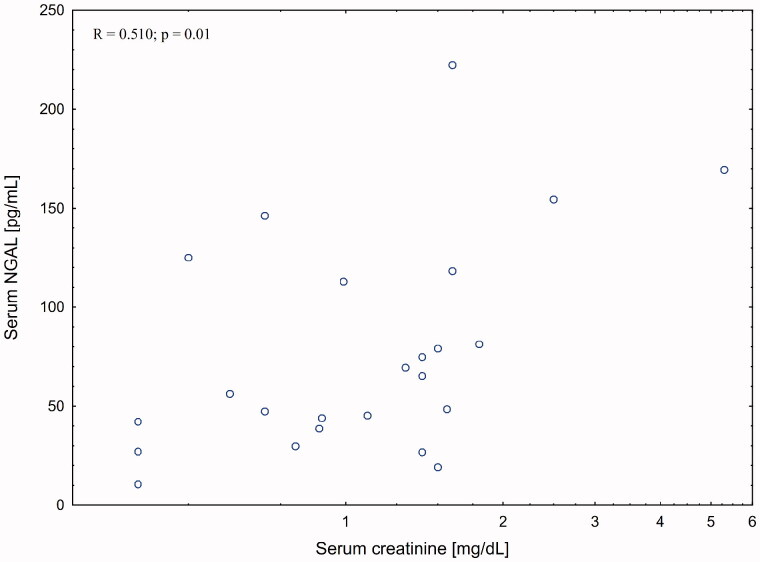
Correlation between serum NGAL and serum creatinine post-paracentesis

**Figure 3.**
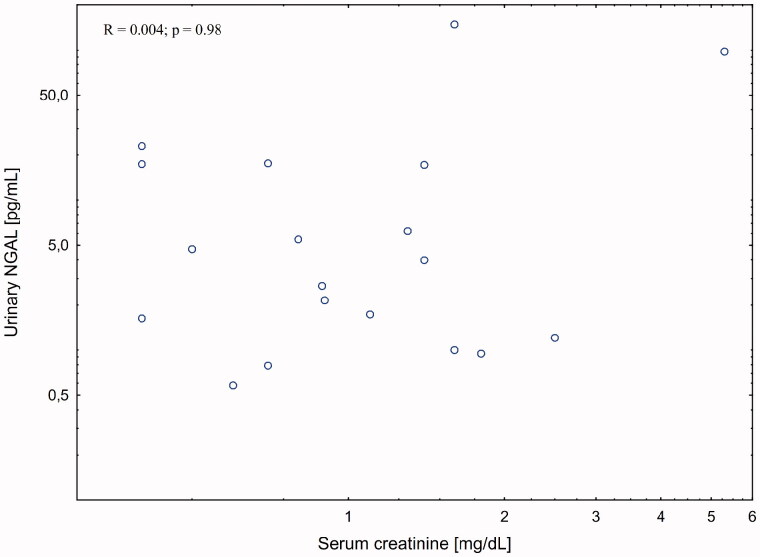
Correlation between urinary NGAL and serum creatinine post-paracentesis.

**Figure 4.**
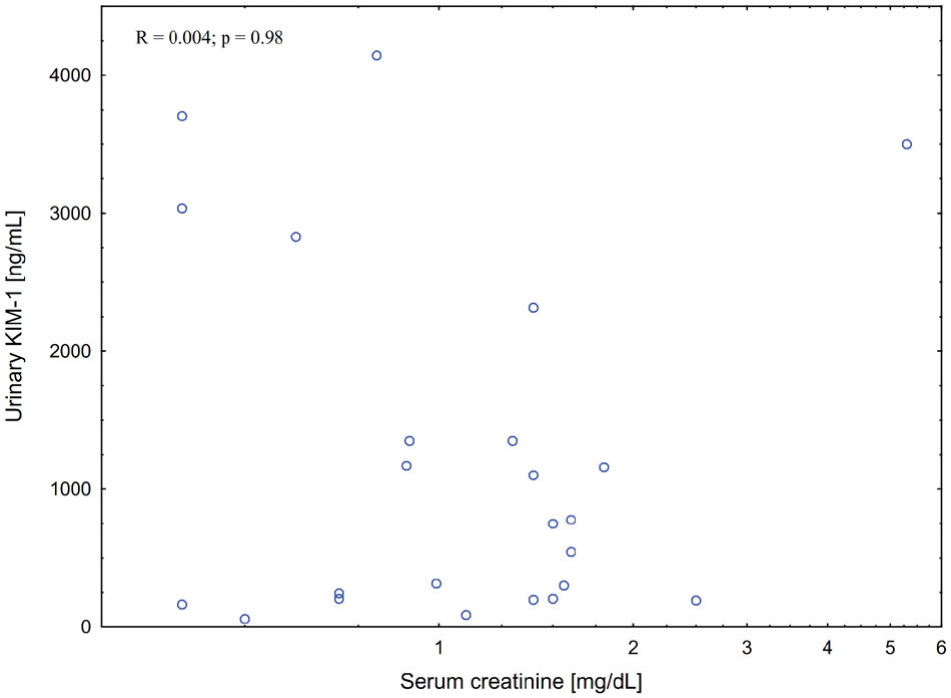
Correlation between urinary KIM-1 and serum creatinine post-paracentesis.

There was no significant correlation between post-procedural changes in serum creatinine and changes in either serum and urinary NGAL or urinary KIM-1 levels (Figures 2–4).

## Discussion

In cirrhosis, both renal impairment and ascites result from the circulatory dysfunction characterized by splanchnic arterial vasodilatation and results in a reduced effective arterial blood volume. Thus, the most important cause of kidney injury in cirrhosis is pre-renal injury, that may be additionally caused by large-volume paracentesis [20]. There is a group of cirrhotic patients, that despite the normal basal kidney function, close monitoring and the proper conservative treatment develop AKI after paracentesis. In the present study, liver cirrhotic patients had near statistically significantly lower eGFR values than the controls. Before paracentesis, the levels of serum and urinary NGAL were similar; while urinary KIM-1 was markedly elevated in liver cirrhotic patients when compared to the controls. All cirrhotic patients had one paracentesis (median paracentesis volume of 5.1 L) with the deterioration of kidney excretory function that met criteria of AKI observed in one-third of them. The subgroup with AKI was characterized by not significantly larger paracentesis volume (6.0 vs 4.8 L). Post-procedure serum NGAL level was 3-times higher in the AKI subgroup, and the difference was statistically significant, however, in the non-AKI group the values were stable. Urinary NGAL and KIM-1 values were stable in the second examination in both AKI and non-AKI subgroups. We did not observe any significant correlation between post-procedural changes in serum creatinine and changes in either serum and urinary NGAL or urinary KIM-1 levels.

Some studies were evaluating the usefulness of both urinary and serum biomarkers for the detection of AKI in patients with chronic liver failure. However, they were quite different than our study. Jiang et al. evaluated the AKI in acute-on-chronic liver failure, but they excluded patients with liver cirrhosis [21]. The other authors, such as Yap et al., Ariza et al., and Jaques et al., studied the utility of several biomarkers, including NGAL and KIM-1, in the outcome assessment in liver cirrhosis. In neither of the cited studies, the authors performed paracentesis [22–24]. AKI in the post-paracentesis state has been rarely studied. Patil et al. [25] found that for each 1 L fluid drained during paracentesis, the risk of AKI increased by 1.24 times and the AKI had occurred in 10.9% of cases. It should be noted that the authors performed on average 10 paracenteses (range 1–15) and the median volume of fluid drained per paracentesis was 6 L (range 1 − 20 L). However, the authors did not assess any of the novel markers of kidney injury. Johnson and colleagues noted that the prevalence of AKI was 1.17%, which is much lower than we and Patil et al. found [26]. This may be a result of a less ascites fluid tapped. As previously, the authors did not evaluate the novel markers of kidney injury. Belcher and colleagues evaluated changes of kidney biomarkers, such as NGAL, KIM-1 and interleukin 18, in the differential diagnosis of patients with cirrhosis and AKI. They found them significantly elevated in patients with acute tubular necrosis when compared to patients with both pre-renal azotemia and hepatorenal syndrome [27]. The authors concluded that kidney biomarkers may help to establish which patients with cirrhosis may have a structural injury underlying their AKI. However, it is hard to determine if such biomarkers can improve outcomes in patients with cirrhosis. In their another study, Belcher et al. observed that multiple structural biomarkers of kidney injury were independently associated with progression of AKI and mortality in patients with cirrhosis, but their levels were similar to those without progression and those with progression alone [3]. Finally, Cai et al. performed an observation that aimed to determine whether the predicted value of urine KIM-1 is correlated with renal KIM-1 expression and tissue damage in AKI patients [28]. The authors observed a close relationship between the severity of renal histological damage and the urine KIM-1 level. The more, in authors’ opinion, combining both urine and serum KIM-1 levels can increase the sensitivity and specificity of the diagnosis of acute renal failure, especially in ATI’s patients.

To summarize all the observations above, one may say that predicting which patients will suffer progressive AKI is still a challenge. On the other hand, the injury markers may serve to identify patients at a highest risk for the worst outcomes who may benefit from early intervention. According to Belcher et al., the likelihood of progression and death is progressively higher with the increasing number of elevated biomarkers. As such, their elevation may precede the deterioration of the patient’s clinical status. In our study, we observed changes only in serum NGAL and KIM-1, associated with the increase in serum creatinine levels post paracentesis. This may be caused by a low number of participants. However, as previously reported by other authors in a larger number of participants (*n* = 188), levels of kidney injury markers were comparable between patients with and without progression of AKI, even though the elevation was associated with poorer prognosis [3].

Our study has limitations. The main limitation is the relatively small group of patients which may lead to a type 2 statistical error, and that the cause of AKI was not clearly defined. However, we do believe, that the main mechanism for AKI was the prerenal azotemia. Our study has some strengths; it was prospective, we utilized both serum and urinary markers, and the patient’s inclusion criteria were tightly specified (it was obligatory for inclusion that patients undergoing paracentesis had at baseline a normal urine output of > 500 mL/day and serum creatinine of ≤ 1.5 mg/dl).

In conclusion, kidney injury markers may be useful for the early detection of AKI and are more sensitive than serum creatinine levels. However, further research in a large cohort is required to determine if biomarkers of kidney injury may help identify patients with cirrhosis who would most likely benefit from early AKI prevention and treatment.

## References

[1] Wong F. Recent advances in our understanding of hepatorenal syndrome. Nat Rev Gastroenterol Hepatol. 2012;9(7):382–391. 22614754 10.1038/nrgastro.2012.96

[2] Sola E, Gines P. Assessment of acute kidney injury at hospital admission in cirrhosis: estimating baseline serum creatinine is not the answer. Liver Int. 2015;3:2079–2081. 10.1111/liv.12883 26053461

[3] Belcher J, Garcia-Tsao G, Sanyal A, et al. Urinary biomarkers and progression of AKI in patients with cirrhosis. Clin J Am Soc Nephrol. 2014;9(11):1857–1867. 25183658 10.2215/CJN.09430913 PMC4220770

[4] Tandon P, Garcia-Tsao G. Renal dysfunction is the most important independent predictor of mortality in cirrhotic patients with spontaneous bacterial peritonitis. Clin Gastroenterol Hepatol. 2011;9(3):260–265. 21145427 10.1016/j.cgh.2010.11.038 PMC3713475

[5] Belcher J, Garcia-Tsao G, Sanyal A, et al. Association of AKI with mortality and complications in hospitalized patients with cirrhosis. Hepatology. 2013;57(2):753–762. 22454364 10.1002/hep.25735 PMC3390443

[6] de Carvalho J, Villela-Noguiera C, Luiz R, et al. Acute kidney injury network criteria as a predictor of hospital mortality in cirrhotic patients with ascites. J Clin Gastroenterol. 2012;46(3):e21–e26. 21934526 10.1097/MCG.0b013e31822e8e12

[7] Jo S, Yang J, Hwang S, et al. Role of biomarkers as predictors of acute kidney injury and mortality in decompensated cirrhosis. Sci Rep. 2019;9(1):14508. 31601879 10.1038/s41598-019-51053-8 PMC6787185

[8] Ichimura T, Bonventre J, Bailly V, et al. Kidney injury molecule-1 (KIM-1), a putative epithelial cell adhesion molecule containing a novel immunoglobulin domain, is up-regulated in renal cells after injury. J Biol Chem. 1998;273(7):4135–4142. 9461608 10.1074/jbc.273.7.4135

[9] Han W, Bailly V, Abichandani R, et al. Kidney injury molecule-1 (KIM-1): a novel biomarker for human renal proximal tubule injury. Kidney Int. 2002;62(1):237–244. 12081583 10.1046/j.1523-1755.2002.00433.x

[10] Liang X, Shi W. Beyond early diagnosis: prognostic biomarkers for monitoring acute kidney injury. Hong Kong J Nephrol. 2010;12(2):45–49.

[11] Yang J, Goetz D, Li JY, et al. An iron delivery pathway mediated by a lipocalin. Mol Cell. 2002;10(5):1045–1056. 12453413 10.1016/s1097-2765(02)00710-4

[12] Bolignano D, Donato V, Lacquaniti A, et al. Neutrophil gelatinase-associated lipocalin (NGAL) in human neoplasias: a new protein enters the scene . Cancer Lett. 2010;288(1):10–16. 19540040 10.1016/j.canlet.2009.05.027

[13] Mishra J, Ma Q, Prada A, et al. Identification of neutrophil gelatinase-associated lipocalin as a novel early urinary biomarker for ischemic renal injury. J Am Soc Nephrol. 2003;14(10):2534–2543. 14514731 10.1097/01.asn.0000088027.54400.c6

[14] Schmidt-Ott KM, Mori K, Li JY, et al. Dual action of neutrophil gelatinase-associated lipocalin. J Am Soc Nephrol. 2007;18(2):407–413. 17229907 10.1681/ASN.2006080882

[15] Lindsay A, Burton J, Ray C. Jr. Paracentesis-induced circulatory dysfunction: a primer for the interventional radiologist. Semin Intervent Radiol. 2014;31(3):276–278. 25177092 10.1055/s-0034-1382799 PMC4140947

[16] Gines P, Tito L, Arroyo V, et al. Randomized comparative study of therapeutic paracentesis with and without intravenous albumin in cirrhosis. Gastroenterology. 1988;94(6):1493–1502. 3360270 10.1016/0016-5085(88)90691-9

[17] Wiesner R, McDiarmid S, Kamath P, et al. MELD and PELD: application of survival models to liver allocation. Liver Transpl. 2001;7(7):567–580. 11460223 10.1053/jlts.2001.25879

[18] Levey A, Stevens L, Schmid C, et al. A new equation to estimate glomerular filtration rate. Ann Intern Med. 2009;150(9):604–612. 19414839 10.7326/0003-4819-150-9-200905050-00006 PMC2763564

[19] Mehta R, Kellum J, Shah S, et al. Acute kidney injury network: report of an initiative to improve outcomes in acute kidney injury. Crit Care. 2007;11(2):R31 17331245 10.1186/cc5713 PMC2206446

[20] Sola-Vera J, Minana J, Ricart E, et al. Randomized trial comparing albumin and saline in the prevention of paracentesis-induced circulatory dysfunction in cirrhotic patients with ascites. Hepatology. 2003;37(5):1147–1153. 12717396 10.1053/jhep.2003.50169

[21] Jiang Q, Han MF, Ma K, et al. Acute kidney injury in acute-on-chronic liver failure is different from in decompensated cirrhosis. WJG. 2018;24(21):2300–2310. 29881239 10.3748/wjg.v24.i21.2300 PMC5989244

[22] Yap D, Wai K, Fung J, et al. Serum and urinary biomarkers that predict hepatorenal syndrome in patients with advanced cirrhosis. Dig Liver Dis. 2017;49(2):202–206. 27876501 10.1016/j.dld.2016.11.001

[23] Ariza X, Sola E, Elia C, et al. Analysis of a urinary biomarker panel for clinical outcomes assessment in cirrhosis. PLOS One. 2015; 10(6):e0128145. 26042740 10.1371/journal.pone.0128145 PMC4456079

[24] Jaques D, Spahr L, Berra G, et al. Biomarkers for acute kidney injury in decompensated cirrhosis: a prospective study. Nephrology (Carlton). 2019;24(2):170–180. 29369449 10.1111/nep.13226

[25] Patil V, Jain M, Venkataraman J. Paracentesis-induced acute kidney injury in decompensated cirrhosis – prevalence and predictors. CEH. 2019;5(1):55–59. 10.5114/ceh.2019.83157 PMC6431093 30915407

[26] Johnson K, Mueller J, Simon T, et al. Reduced albumin dosing during large-volume paracentesis is not associated with adverse clinical outcomes. Dig Dis Sci. 2015;60(7):2190–2195. 25724164 10.1007/s10620-015-3578-z

[27] Belcher J, Sanyal A, Peixoto A, et al. Kidney biomarkers and differential diagnosis of patients with cirrhosis and acute kidney injury. Hepatology. 2014;60(2):622–632. 24375576 10.1002/hep.26980 PMC4065642

[28] Cai J, Jiao X, Luo W, et al. Kidney injury molecule-1 expression predicts structural damage and outcome in histological acute tubular injury. Ren Fail. 2019;41(1):80–87. 30909833 10.1080/0886022X.2019.1578234 PMC6442099

